# An Interdisciplinary Approach to the Management of Ketamine Induced Uropathy

**DOI:** 10.1192/bjo.2024.676

**Published:** 2024-08-01

**Authors:** Rebecca McKnight, Patrick Horgan

**Affiliations:** Greater Manchester Mental Health NHS Foundation Trust, Manchester, United Kingdom

## Abstract

**Aims:**

This report describes the treatment of a patient with ketamine induced uropathy. This condition can be significantly debilitating due to its severe effect on the urinary system. This report outlines an interdisciplinary approach to the care of the patient involving addictions services, urology and primary care.

**Methods:**

The patient presented with a history of inhalation of ketamine intermittently for four years and daily for three years. His highest daily use was 14 grams per day.

He developed multiple urinary symptoms including dysuria, urgency, incontinence, haematuria and abdominal and urethral pain. He had significant weight loss and suicidal thoughts. After six years of use, he was reviewed by urology, however was discharged within a year, after missing appointments. Investigations included ultrasound which showed kidneys of normal appearance; flexible cystoscopy which showed a small bladder with acute bleeding and posterior wall ulcer; urodynamic studies showed overactive bladder.

He attended a private, inpatient detoxification programme, however relapsed after this admission and self-referred to local addictions services. When assessed there, a detailed history and physical examination were completed. Baseline electrocardiogram and blood investigations were completed and were broadly normal.

It was felt that a collaborative approach between addictions services, primary care, urology, and a regional addictions detoxification centre could help him manage his symptoms and achieve more sustained abstinence. Following interdisciplinary discussions, he was commenced on solifenacine to treat his urinary frequency, mirtazapine for his mood and buscopan for pain. Motivation interviewing approaches were used to help him reduce his ketamine use.

**Results:**

Ketamine is a synthetic drug with marked dissociative, stimulant and hallucinogenic properties. There has been a rising trend in adults entering treatment with harmful ketamine use in recent years. In 2023, 2,211 people entered treatment for harmful ketamine use in England, a fivefold increase from 2014. Ketamine induced uropathy would be expected to occur in a high proportion of these people. A survey of adolescents demonstrated that 60% of ketamine users had lower urinary tract symptoms. There are a range of medical and surgical options to treat ketamine induced uropathy but no clear agreed approach for its holistic management in the UK.

**Conclusion:**

This case report highlights the consequences of prolonged ketamine use on the urinary tract system. It highlights an example of effective interdisciplinary working between addictions services, primary care and urology. The authors recommend the development of nationally agreed guidelines on ketamine induced uropathy with emphasis on collaborative, inter-service working.

